# The role of Neurochemicals, Stress Hormones and Immune System in the Positive Feedback Loops between Diabetes, Obesity and Depression

**DOI:** 10.3389/fendo.2023.1224612

**Published:** 2023-08-17

**Authors:** Julian B. Wilson, Ma’ayan Epstein, Briana Lopez, Amira K. Brown, Kabirullah Lutfy, Theodore C. Friedman

**Affiliations:** ^1^Department of Internal Medicine, Charles R. Drew University of Medicine and Science, Los Angeles, CA, United States; ^2^Psychiatric Emergency Room, Olive View – University of California, Los Angeles (UCLA) Medical Center, Sylmar, CA, United States; ^3^Friends Research Institute, Cerritos, CA, United States; ^4^College of Pharmacy, Western University of Health Sciences, Pomona, CA, United States

**Keywords:** diabetes, depression, obesity, dopamine, serotonin, lifestyle medicine, monoamines

## Abstract

Type 2 diabetes mellitus (T2DM) and depression are significant public health and socioeconomic issues. They commonly co-occur, with T2DM occurring in 11.3% of the US population, while depression has a prevalence of about 9%, with higher rates among youths. Approximately 31% of patients with T2DM suffer from depressive symptoms, with 11.4% having major depressive disorders, which is twice as high as the prevalence of depression in patients without T2DM. Additionally, over 80% of people with T2DM are overweight or obese. This review describes how T2DM and depression can enhance one another, using the same molecular pathways, by synergistically altering the brain’s structure and function and reducing the reward obtained from eating. In this article, we reviewed the evidence that eating, especially high-caloric foods, stimulates the limbic system, initiating Reward Deficiency Syndrome. Analogous to other addictive behaviors, neurochemical changes in those with depression and/or T2DM are thought to cause individuals to increase their food intake to obtain the same reward leading to binge eating, weight gain and obesity. Treating the symptoms of T2DM, such as lowering HbA1c, without addressing the underlying pathways has little chance of eliminating the disease. Targeting the immune system, stress circuit, melatonin, and other alterations may be more effective.

## Introduction

It has been over 30 years since Reaven first described metabolic syndrome, which commonly affects overweight individuals (1). Obesity has become endemic in the developed world and is on its way to becoming so in developing nations, producing many health-related problems (2). Not only is weight loss difficult to achieve, but those who lose weight also find keeping it off is even more difficult (3), causing many to propose that food is addictive. Over the years, evidence in support of this has accumulated, and reliable scales to diagnose “eating addiction” (also known as “food addiction”) have been generated and used to assess its prevalence in the population (4).

Work on the neuronal basis of eating addiction has also been conducted. Traditionally this has involved studying both central and peripheral molecules involved in hunger and satiety, such as leptin, orexin (also known as hypocretin), insulin, alpha-melanocyte-stimulating hormone (α-MSH), glucagon-like peptide -1 (GLP-1), amylin, glucose-dependent insulinotropic polypeptide (GIP, also known as a gastric inhibitory polypeptide), adiponectin and cholecystokinin (CCK). However, it is well known in psychiatry that neurotransmitters are also involved. Soon after the introduction of atypical antipsychotics, which antagonize serotonin receptors and dopamine D_2_ receptors (D_2_R), numerous case reports appeared showing that the use of these drugs were associated with increased obesity and the development of type 2 diabetes mellitus (T2DM) (5). Conversely, the D_2_R agonist bromocriptine, which has been used for over 40 years to treat Parkinson’s disease and hyperprolactinemia (6), was found to lower blood glucose levels and improve insulin sensitivity in patients with T2DM (7). In 2009, the US Food and Drug Administration (FDA) approved a rapid-release bromocriptine formulation (Cycloset™) to treat T2DM (8). These observations demonstrate that hunger and satiety, and energy homeostasis, are controlled by dopamine and serotonin signaling. Increasing dopamine levels/signals are associated with improved insulin sensitivity, while decreasing dopamine and serotonin levels/signals are associated with weight gain and T2DM development.

**Figure 1:**
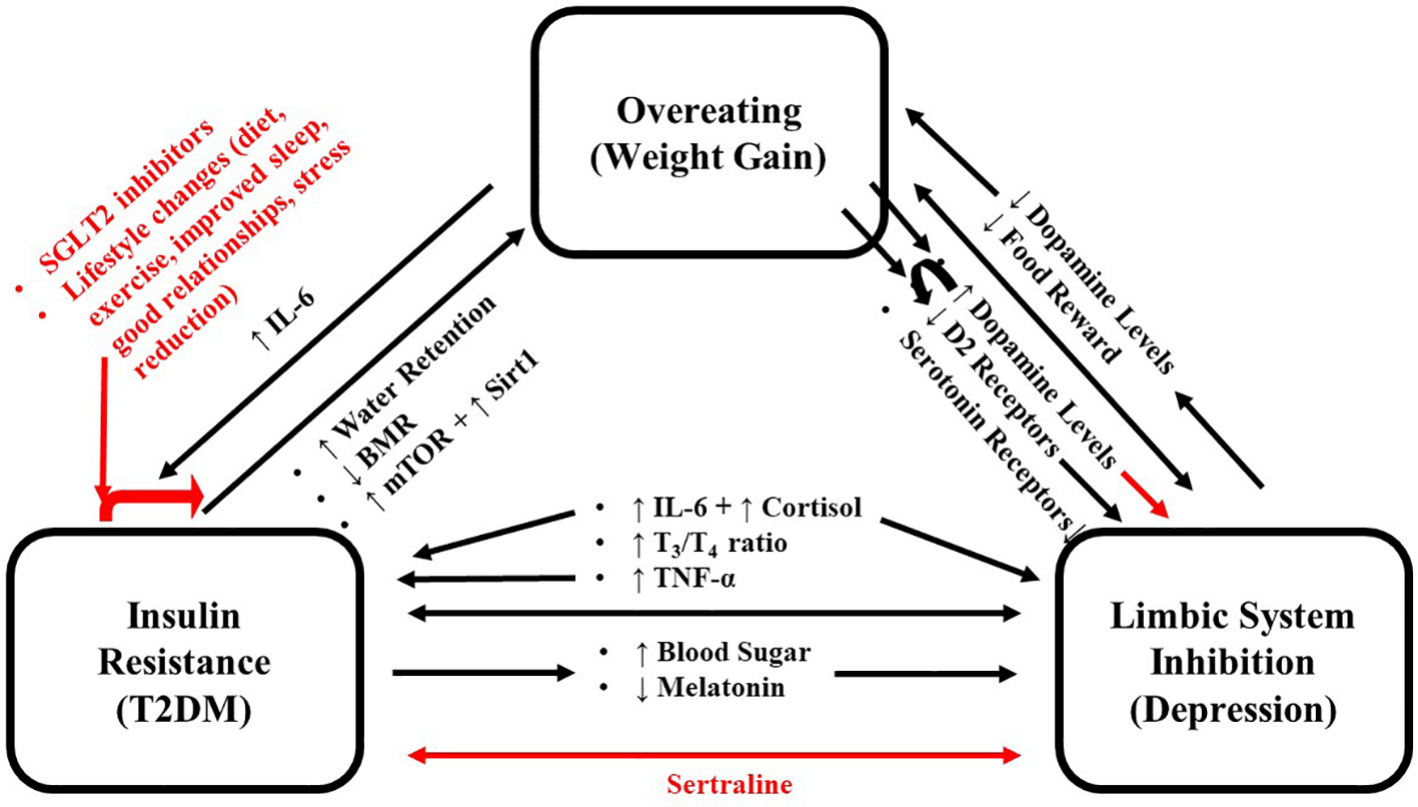
Schema for the positive feedback loop where overeating, limbic system inhibition and T2DM reinforce each other. Black arrows indicate promoting effects, and red arrows indicate inhibitory effects. IL-6, Interleukin 6; T2DM, Type 2 Diabetes Mellitus.

The above research focused on the effects of food intake on serotonin and dopamine separately. However, in the limbic system, their levels are coupled and modulated by changes in this group of interconnected structures that together regulate emotional and motivated behaviors (such as reward) (9). Like other addictive behaviors, eating over time can inhibit the limbic system and reduce the reward attained from eating (10). This inhibition can cause individuals to eat more to obtain the same level of pleasure/satiety, leading to weight gain (11). This represents a cycle whereby eating/weight gain and limbic system inhibition reinforce and strengthen one another contributing to increased body weight and obesity (12). The resulting weight gain is also associated with developing T2DM; contributing to the metabolic changes associated with this disease (13). The mild hypercortisolism associated with T2DM (14–16) could also increase appetite and inhibit sleep, leading to weight gain (17). T2DM decreases metabolic rate *via* the release of the stress hormone, cortisol, and inflammatory markers, such as interleukin-6 (IL-6) (18). Cortisol and IL-6 are important in the pathogenesis and maintenance of both T2DM and depression (a state of extreme limbic system inhibition) (19, 20). T2DM can also cause weight gain indirectly *via* inhibition of the limbic system (discussed below). Hyperglycemia is neurotoxic and causes limbic system inhibition (10, 21), showing that overeating/weight gain does not induce one, but three feedback cycles where overeating, limbic system inhibition, and T2DM reinforce and strengthen one another, which expose individuals to further weight gain and enhance the negative impact of T2DM and limbic system inhibition (**Figure 1**). This review summarizes the various molecular mechanisms that set the stage for these cycles and includes recommendations to abort these cycles. Although multiple mechanisms have been proposed for the association between depression and diabetes, we chose to focus on the immune system and HPA axis for several reasons as follows: The first being that these are the most studied and provide a clearly observable direct link between the two which can be explained using evolutionary arguments. The second reason is that they can be used to mechanistically categorize “typical” depression (melancholic, which is associated with both increased immune system and HPA activation) and “atypical” depression (which is associated with increased immune system but normal HPA activation).

## Food and addiction: serotonin and dopamine

In order to maintain physiological homeostasis, eating is a rewarding behavior (11), that acts on the brains pathways similarly to other rewards and sometimes leads to an eating addiction. Serotonin and dopamine are important neurochemicals in regulating mood and reward, respectively. Like other addictive substances, food directly affects the release of these molecules (22). It has been known for nearly 40 years that brain serotonin levels are inversely proportional to both food intake and body weight, and many of the mechanisms behind this have been well elucidated (23). The serotonin 5-HT2C receptor has been particularly implicated in this (24). Deficiency of this receptor or its polymorphism leads to increased food intake and obesity through dysregulation of the proopiomelanocortin (POMC)-containing neurons (24, 25). These hypothalamic neurons undergo neurogenesis into adulthood and regulate metabolism and reproduction (24, 26). Conversely, it has also been shown that serotonin 5-HT2C receptor agonists (such as m-chlorophenylpiperazine) improve insulin sensitivity (27).

Analogous studies have been carried out with dopamine, where dopamine D2R is implicated in regulating food intake and addictive behaviors (28). Dopamine is required for feeding in mice (29), where there is an inverse correlation between body mass index (BMI) and striatal D2R (30). Loss of this receptor leads to insulin resistance (31). In humans, it has been shown that expression of the D2R is reduced in obese individuals (32), and activation of this receptor alleviates many aspects of metabolic syndrome in obese women (33). Variations of the D2R allele, although rare in the general population, have a higher prevalence in obese individuals, those with alcoholic and tobacco use disorders, and people who use other addictive substances (34–36).

Food consumption alters the mesolimbic dopaminergic neuronal activity (28), one of the four dopaminergic pathways in the brain, which plays a key role in reward and motivation (20). Like other addictive substances, food consumption triggers dopamine release in the nucleus accumbens (also known as the ventral striatum) and dorsal striatum (11). In the dorsal striatum, the amount of dopamine released is proportional to the palatability of the meal (37). Alterations to the mesolimbic pathway, either by genetic approaches or *via* pharmacological tools, affect both feeding and addictive behaviors. Furthermore, fasting increases the rewarding effects of both food (38) and addictive substances (39), while addictive substances have been shown to alter the expression of genes involved in food intake (40) and increase the response to food intake in the hypothalamus (41).

As with other addictive substances, there is evidence that obese individuals develop a tolerance to food reward (11, 32), setting the stage for overeating and addiction. In a condition known as Reward Deficiency Syndrome (RDS), which is compensated by overeating to restore hedonic homeostasis, the exaggerated dopamine release from eating leads to a reduction in D2R density and altered dopamine signaling during subsequent meals, which causes the individual to eat more to obtain the same level of satiety (11). This further reduces the satiety obtained from eating and may initiate a vicious cycle, driving individuals towards overeating and obesity. Consistent with this, weight gain has been shown to reduce striatal activation in response to food intake (42).

## The limbic system and depression

The limbic system controls brain-wide monoamine levels, of which four brain regions are particularly significant. The prefrontal cortex facilitates executive functions by orchestrating the operations of other brain regions. It has extensive connections to various hypothalamic nuclei to monitor and control autonomic functions and energy homeostasis (43). The amygdala, traditionally associated with anxiety and fear conditioning, is also involved in positive and negative affective states, attention, and reinforcement and directly controls autonomic and endocrine manifestations of fear and stress (44). The hippocampus, which plays a vital role in learning and memory, also plays a vital role in spatial representation (45). The hippocampus and the olfactory bulb are the two brain areas that participate in life-long neurogenesis in adults (46, 47). The hypothalamus receives input from the other limbic structures and modulates physiological processes accordingly. Importantly, it contains neurons that secrete corticotropin-releasing hormone (CRH, also known as corticotropin-releasing factor) which stimulates adrenocorticotropic hormone (ACTH) secretion from the pituitary gland, which in turn stimulates cortisol secretion from the adrenal gland. This pathway is collectively known as the hypothalamic-pituitary-adrenal (HPA) axis, which mediates the stress response. These CRH-producing neurons generally receive stimulatory input from the amygdala, while the prefrontal cortex and hippocampus send inhibitory inputs to these cells (48, 49).

Collectively, the limbic system receives sensory (from the external environment) and autonomic (from inside the body) inputs and acts on a collection of neuromodulatory neurons in the locus coeruleus (where the cell bodies of norepinephrine-producing neurons are located), raphe nuclei (where the cell bodies of serotonin-producing neurons are located), the ventral tegmental area and the substantia nigra pars compacta (where the cell bodies of dopamine-producing neurons are located), the basal forebrain (where the cell bodies of acetylcholine-producing neurons are located), and the tuberomammillary nucleus (where the cell bodies of histamine-producing neurons are located). The neurons in these locations project widely throughout the brain to modulate excitability, to control the “state” of mind, which can be considered emotions (50). Individuals with depression have reduced mood, and pharmacological interventions used to treat depression primarily work by boosting monoamine levels (51). Similarly, monoamine depletion has been shown to decrease mood in individuals with a personal or a family history of depression. However, it did not affect individuals who lack these risk factors (52). Finally, several studies have demonstrated a link between genetic polymorphisms in the serotonergic pathway and depression (20). These findings suggest that depression is brought about, in part, by the diminished activity of the limbic system.

In addition to depressed mood, individuals with depression are unaffected by natural reward, known as anhedonia, which could initiate RDS (53). Taken together, depression is an important risk factor in obesity and addictive behavior (54, 55).

## Depression causing insulin resistance and T2DM: glucocorticoids and IL-6

There is an intimate connection between the HPA axis and the immune system. Depression typically involves a dysregulated positive feedback loop between these two systems (20), as opposed to atypical depression, where the HPA axis activity is normal or decreased (56). The HPA axis and the immune system can act synergistically to cause insulin resistance, leading to T2DM. The interaction between the HPA axis and the immune system is complex. Although it is well known that chronically elevated glucocorticoid levels suppress the immune system, acute elevation in stress hormones stimulates the immune system, and basal glucocorticoid levels are required for immune system activation (57). Similarly, products of inflammation stimulate the HPA axis, most notably proinflammatory cytokines (described below). The usual interaction between the HPA axis and immune system breaks down in individuals with depression, where the levels of both glucocorticoids and inflammatory markers are elevated (20).

There may be an evolutionary basis for depressive behaviors and the underlying mechanisms that maintain them. It has been suggested that depression arose from the need to control metabolism during stress periods (58). When activated, the immune system requires roughly 25 – 30% of the basal metabolic rate to fight infections (58). To provide this, the immune system and the HPA axis both act on the brain to induce “sickness behaviors” such as increased sleep, fatigue, internal focus, and anhedonia (decreased reactivity to natural reward) to limit the energy demands of the organism (20). HPA axis activation leads to glucocorticoid secretion from the adrenal glands, chiefly cortisol, in humans. Glucocorticoids are lipid-soluble and can freely cross the blood-brain barrier to act on mineralocorticoid and glucocorticoid receptors expressed in discrete brain areas, particularly in the limbic areas and the pituitary gland (59). Elevated glucocorticoid levels, especially in depression, increase blood flow and glucose metabolism in the amygdala (60, 61). In depressed individuals, amygdala activation correlates with both depression severity and the dispositional negative affect (61). In the prefrontal cortex and hippocampus, which have the highest glucocorticoid and mineralocorticoid receptor levels in the brain (62), elevated glucocorticoid levels induce cellular atrophy and death. This results in a reduction in both brain activity and grey matter volume (63, 64). Considering these areas also modulate HPA axis activity, glucocorticoid release can initiate a detrimental positive feedback loop, where glucocorticoid-mediated stimulation of the amygdala (which generally stimulates the HPA axis) and inhibition of the prefrontal cortex and hippocampus (which generally send inhibitory input to the HPA axis) leads to progressively increased and ultimately dysregulated HPA axis activation. This HPA axis dysregulation, and the resulting increase in glucocorticoid levels, is important to the induction and maintenance of depression and is comparable to that observed in Cushing’s disease (20), in which a small group of dysregulated cells in the pituitary gland (an adenoma) secretes adrenocorticotropic hormone, leading to hypercortisolism.

## The HPA axis and the immune system in depressive behaviors

The immune system can directly stimulate the HPA axis and induce depressive behaviors. Proinflammatory cytokines like tumor necrosis factor-α (TNF-α), interleukin (IL)-1β, and IL-6 act on the brain to stimulate the HPA axis and induce behaviors that conserve energy (sickness behaviors) (20, 58). Numerous mechanisms exist for the latter, including directly activating neurons or the blood-brain barrier endothelial cells and the ability of IL-1β to induce the serotonin transporter expression (resulting in reduced synaptic serotonin levels) (52, 65). However, particular focus has been given to indoleamine 2,3 dioxygenase (IDO) activation. Under normal conditions, the liver degrades over 95% of dietary tryptophan, with the remainder used for serotonin synthesis and other functions (66). Under inflammatory conditions, cytokines activate IDO to degrade tryptophan through the kynurenine pathway in the central nervous system (CNS) and peripherally, where it plays a role in nicotinamide adenine dinucleotide (NAD) synthesis and dampens the immune response to prevent autoimmune disease development (a process known as a peripheral tolerance) (67). This depletes serotonin levels in the CNS and leads to dysregulated neurotransmission to the point of neuronal damage since many of the kynurenine pathway metabolites are neuroactive. The kynurenine pathway produces products such as quinolinic acid (an agonist of the N-methyl-D-aspartate receptors, NMDARs) and kynurenic acid (which activates both glutamate and nicotinic receptors), which have been implicated in depression as well as other psychiatric and neurodegenerative disorders (68, 69).

This association between cytokines and depression has been demonstrated clinically. Patients with inflammatory diseases have a higher prevalence of depression (70), and anti-inflammatory therapies have been shown to reduce depressive symptoms independent of changes in disease symptoms (71). This was best exemplified by one study where individuals injected with the *Salmonella typhi* vaccine demonstrated a strong correlation between the induction of negative mood and IL-6 production (72). Psychological stress has been shown to increase the production of inflammatory cytokines IL-1β, IL-6, and TNF-α (73), and depressed individuals have been found to have elevated levels of proinflammatory cytokines (68). These observations demonstrate the important role of proinflammatory cytokines, notably IL-6, in the induction and maintenance of depression.

The immune system (via proinflammatory cytokines) and the HPA axis (via glucocorticoids) can contribute to the prevalence of metabolic syndrome and the resulting obesity observed in depression (74). Depressed individuals have HPA dysfunction, analogous, but to a lesser degree, to those with Cushing syndrome, a condition referred to as Pseudo-Cushing syndrome (14), which can be observed in other conditions, including alcoholism, T2DM and PCOS. Patients with depression can have enlarged adrenal glands (75), elevated plasma cortisol levels (19), and altered circadian cortisol secretion rhythms that fail to be suppressed by dexamethasone (76, 77). Similarly, we have shown that the high cortisol levels, as seen in Cushing’s syndrome, cause insulin resistance and alter lipid metabolism analogous to that observed in T2DM (78). Glucocorticoids also stimulate food consumption, particularly low-quality foods (79, 80), far above the modest energy increase, and in spite of increased levels of the satiety hormone leptin (81). Glucocorticoids promote muscle wasting (which lowers the body’s basal metabolic rate) and promote adipogenesis with a preference for central obesity (82, 83). This is due to increased expression of glucocorticoid receptor and 11β-hydroxysteroid dehydrogenase type 1, which converts inactive 11-dehydrocorticosterone (cortisone) to active cortisol to amplify glucocorticoid signaling (84, 85). Glucocorticoids also stimulate catecholamine synthesis and release. Catecholamines can stimulate glucagon from the pancreas, and both act on the liver to potentiate glycogenolysis, gluconeogenesis, and thus the hepatic release of glucose into the bloodstream leading to hyperglycemia (86). High levels of glucocorticoids and inflammatory cytokines have been shown to reduce thyroid hormone production as well as reduce the conversion of thyroxine (T_4_) to 3,5,3′-triiodo-L-thyronine (T_3_), setting up a condition called nonthyroidal illness (euthyroid sick syndrome) (87, 88). Patients with this condition have low thyroid hormones without signs and symptoms of hypothyroidism (89). Cortisol also acts as a mineralocorticoid and is responsible for half of our daily mineralocorticoid activity (90), so conditions that elevate plasma cortisol levels may lead to fluid retention and weight gain (91). These data show that the glucocorticoids and inflammatory cytokines seen in depression can cause weight gain.

Proinflammatory cytokines also antagonize insulin signaling through various mechanisms, contributing to the onset of T2DM. TNF-α inhibits insulin signaling by phosphorylating serine residues on the insulin receptor substrate-1 (IRS-1) protein, inhibiting insulin-induced tyrosine phosphorylation (92, 93). The IL-6 receptor is a class I cytokine receptor, like the insulin receptor (90), and uses the Janus kinases/signal transducers and activators of transcription (JAK/STATs) molecules. Its activation has been shown to impair insulin signaling either directly or *via* suppressor of cytokine signaling (SOCS) proteins (94). Importantly, although insulin-resistant adipocytes have been shown to increase expression of TNF-α, IL-6, and IL-8, only IL-6 released into the systemic circulation induces insulin resistance in the liver and the production of C-reactive protein (95, 96). Elevated levels of this protein have been associated with cardiovascular complications and a two-fold increase in the risk for T2DM within 3 – 4 years (97–99). These findings show that the inflammatory cytokines present in depression contribute to the pathogenesis of depression and are a significant consequence of T2DM.

## T2DM causing depressive symptoms: IL-6 and glucocorticoids

It is now understood that obesity is associated with chronic inflammation, with elevated levels of proinflammatory cytokines playing a direct role in insulin resistance (13). Numerous studies in *Drosophila*, rodents, and humans have implicated the cluster of differentiation 36 (CD36) in insulin resistance (100, 101). This class B scavenger receptor is widely expressed and is involved in the clearance of apoptotic cells, pathogens, thrombospondin-1, modified low-density lipoproteins, and long-chain fatty acids (102). Although numerous factors are involved, elevated serum concentrations of lipids, oxidized low-density lipoproteins, or glucose lead to a pathological increase in CD36 expression (101). This is likely caused by inducing a positive feedback loop *via* peroxisome proliferator-activated receptors (PPARs), most notably PPARγ, which senses lipids and oxidized low-density lipoproteins intracellularly and upregulates CD36 expression to increase ligand endocytosis (103). Interestingly, hyperglycemia can also increase CD36 expression both directly and indirectly (100). CD36 activates C-Jun N-terminal kinase (JNK), and both are required for macrophages to adopt a foamy appearance (101). The resulting “foam cells” secrete chemokines such as monocyte chemotactic protein 1 (MCP-1, also known as CCL2) that recruit additional monocytes, enhance CD36 signaling (104), and secrete inflammatory cytokines such as IL-1β, IL-6, and TNF-α (104) that antagonize insulin signaling in adipose tissue (discussed above). Of these, IL-6 is the only cytokine that leaves adipose tissue and is found in the systemic circulation, with its serum concentrations proportional to HbA_1c_ levels (96, 105).

In the liver, IL-6 antagonizes insulin signaling and induces inflammation, which after reaching a certain threshold, leads to insulin resistance and non-alcoholic fatty liver disease (18, 106). In the brain, IL-6 stimulates the HPA axis by acting on both the hypothalamus and the pituitary gland to stimulate the secretion of CRH and ACTH, respectively, leading to glucocorticoid secretion (107, 108). Earlier, we discussed how the subsequent action of glucocorticoids on the liver is important in maintaining T2DM (109, 110). It also activates local inflammatory networks in the brain, which reduce hippocampal neurogenesis, leading to limbic system dysfunction and depressive symptoms such as those associated with energy conservation (68, 111, 112). These observations demonstrate that the pathogenesis of T2DM and depression involve the same pathways (the immune system and HPA axis activation) and the same molecules (IL-6 and cortisol). Thus, both T2DM and depression reinforce and potentially exacerbate one another.

## T2DM causing depression: glucose

In addition to IL-6 and glucocorticoids, the hyperglycemia seen in T2DM can also act on the brain to induce depression and obesity. Glucose can depolarize or hyperpolarize discrete subsets of neurons throughout the brain, particularly in the hypothalamus (113). These neurons are thought to play a role in energy homeostasis, in part, by regulating dopamine release (114). In many of these neurons, the entry of glucose inside the neuron generates energy in the form of ATP, which closes ATP-sensitive potassium channels, analogous to pancreatic β-cells, and is regulated by insulin. In obese rats, the ability of insulin to activate ATP-sensitive K+ channels is lost and can lead to dysregulation in feeding and metabolism (115). Hyperglycemia is also neurotoxic, producing reactive oxygen species, DNA damage, mitochondrial swelling, and apoptosis even at levels found in prediabetes (21). Although numerous mechanisms are involved (21), inhibition of autophagy, reduction of melatonin levels, and atherosclerosis are particularly harmful to neurons.

## Autophagy inhibition: mTOR/Sirt1

Another way that hyperglycemia can cause neuronal damage and dysfunction is through inhibition of autophagy. Autophagy is when a cell destroys abnormal proteins and senescent organelles (particularly mitochondria because of their high turnover rate) in the cytoplasm. The first process involves signaling from the mammalian target of rapamycin (mTOR) and the Sir2-like protein 1 (Sirt1), both involved in cellular metabolism. Their pathways interact with each other through AMP-activated protein kinase (AMPK) and other mechanisms (116). mTOR is a serine/threonine kinase of the PI3K-related kinase family that regulates growth factors and nutrient initiation of transcription, translation, and numerous other cellular functions (117). Sirt1 is a nicotinamide adenine dinucleotide (NAD+) dependent deacetylase that acts on a variety of proteins that have been implicated in cancer and energy homeostasis, including p53, nuclear factor-κβ (NF-κβ), PPAR γ co-activator-1α (PGC-1α), and forkhead transcription factor (FOXO) (118).

Typically, in individuals without T2DM, blood sugar can be elevated during the day (due to eating and the circadian rise in cortisol levels). This inhibits Sirt1 and activates mTOR to initiate protein production and cell growth while inhibiting autophagy (119, 120). At night, these processes are reversed, as sleep prevents eating. Also, there is a circadian-dependent nadir of cortisol at night. This process inhibits mTOR, thereby reducing protein production and removing the inhibition of autophagy. It also increases Sirt1 activity to induce autophagy (119, 120). Autophagy is a highly conserved pathway that removes protein aggregates, unneeded or damaged organelles (particularly mitochondria), and invading pathogens (121, 122). The circadian oscillation in blood sugar levels is critical in protein synthesis during the day and in removing damaged or aggregated proteins at night. This process is particularly important in neurons as they produce large amounts of proteins (such as neurotransmitters) needed for signaling. They also contain long thin processes (dendrites and axons) where these proteins can reach high concentrations and aggregate. The accumulation of protein aggregates is common in most neurodegenerative diseases (123). In agreement with this, autophagy inhibition has been shown to induce protein aggregate formation (124), while autophagy activation removes these aggregates and thus is beneficial in such diseases (125). Additionally, impaired autophagy also leads to impaired mitochondrial clearance and dysfunction. The latter can lead to decreased adenosine triphosphate (ATP) levels as well as increased reactive oxygen species (ROS) production and membrane peroxidation that can impair neuronal signaling and function, leading to mitochondrial dysfunction all of which has been implicated in depression.

Both mTOR and Sirt1 are involved in the pathogenesis of T2DM and are therapeutic targets of the widely used antidiabetic drug metformin (116). Hyperglycemia, especially chronic hyperglycemia observed in T2DM, leads to increased protein production and autophagy inhibition. This, in turn causes neuronal dysfunction while making these neurons more prone to protein aggregate formation and subsequent damage. Since limbic structures project widely (their axons spread throughout the brain) they are particularly at risk to this type of damage, which would lead to overall limbic system inhibition and initiate the RDS. Since autophagy diminishes with age (122), older individuals are more at risk of the pathological impact of hyperglycemia.

## Melatonin and free radical damage

Melatonin is a highly conserved and multifunctional molecule. It evolved around 2.5 billion years ago during the Great Oxygen Event when photosynthetic bacteria first originated and began poisoning the atmosphere with oxygen gas, which was toxic to most life at the time (126). Melatonin, a powerful antioxidant, allowed bacteria to survive in this new atmosphere (127). Since then, organisms have evolved to use this molecule for other functions, such as regulation of the circadian rhythm and reproduction, but it has retained its function as a free radical scavenger and is important in cancer prevention (127). The predominant melatonin source in mammals is the pineal gland, a highly vascularized organ located above the posterior commissure in the roof of the third ventricle. Circadian rhythms and darkness stimulate the synthesis and secretion of melatonin from this organ in a progressive manner, with serum melatonin levels rapidly rising from basal levels of approximately 40 pmol/L (in adult males) to maximum levels of approximately 300 pmol/L (in young adult males) by the middle of the darkness period (127, 128). Melatonin acts on high-affinity G-protein coupled receptors (GPCRs) located on the plasma membranes and also enters the cells primarily through glucose transporters to activate the cytosolic enzyme quinone reductase 2 (a detoxification enzyme), activate nuclear receptors to modulate transcription, and directly scavenge free radicals throughout the cell (127, 129).

Hyperglycemia reduces melatonin production and is partly responsible for the mitochondrial abnormalities and free radical damage observed in T2DM and contributes to neuronal dysfunction (130, 131). The decrease in melatonin and the resulting increase in free radical damage decreases the number of neurons in the hippocampus, contributing to neuronal senescence, damages signaling molecules in plasma membranes, and inhibits proteasomes to cause protein aggregate formation in a manner analogous to autophagy inhibition (discussed above) (132–134). The resulting neuronal dysfunction inhibits the limbic system and alters many neuroendocrine pathways pathologically.

Decreased nighttime melatonin levels have been implicated in depression, bringing about RDS and overeating (discussed above). Melatonin receptor knockout mice display depression-like behaviors (135). In humans, single nucleotide polymorphisms in melatonin receptors have been shown to modulate the lifetime risk of depression (136). Interestingly, melatonin receptor agonists have been shown to be beneficial in treating depression and other depressive mood disorders (136–138). As with autophagy, pineal gland melatonin production decreases with age; therefore, older individuals may be more susceptible to the complications of hyperglycemia (128).

### Impact of T2DM on limbic function and structures

**Figure 2:**
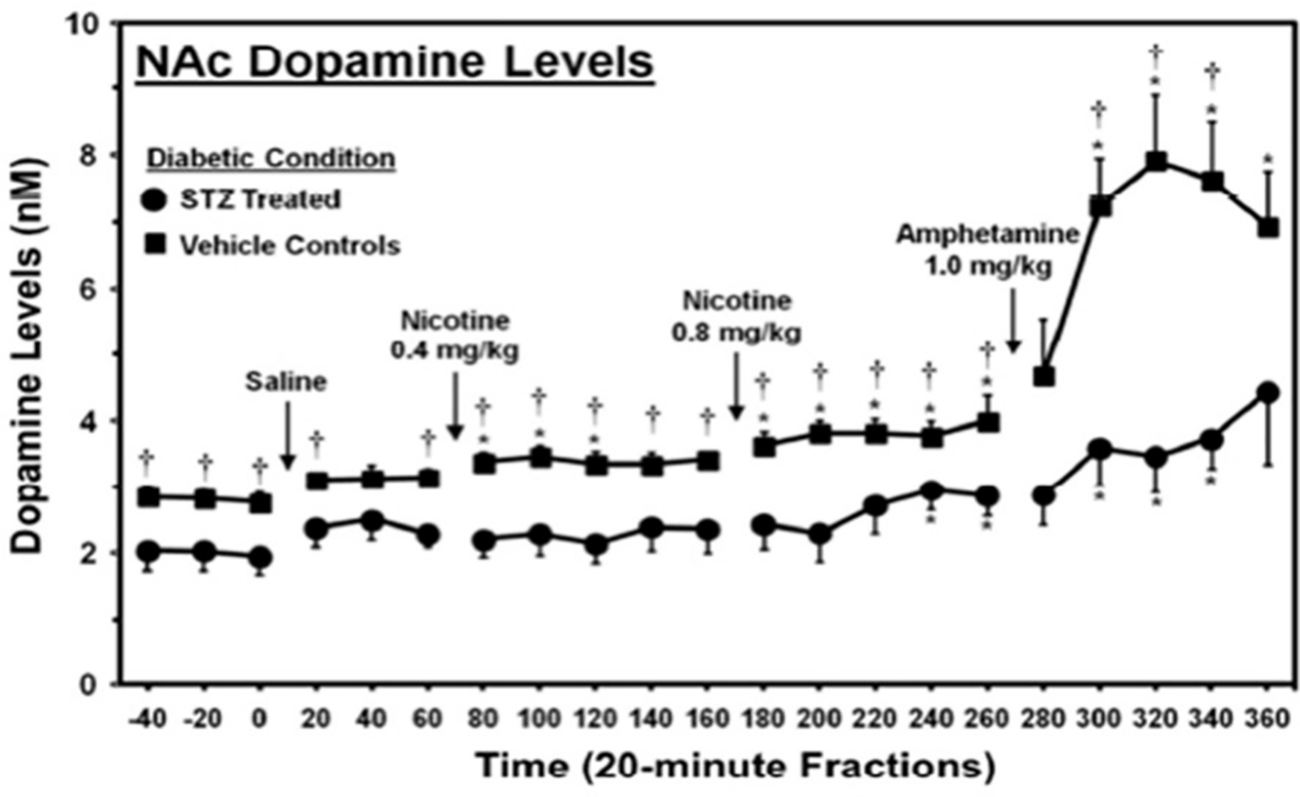
Dopamine levels in the nucleus accumbens of streptozotocin (STZ)- and vehicle-treated control rats. *, denotes a significant difference relative to baseline. †, denotes significant difference between STZ- and vehicle control groups [from (139), permission given by publisher].

The ability of hyperglycemia to inhibit limbic function, leading to RDS and overeating, was demonstrated in an experiment done by O’Dell et al. using rats treated with streptozotocin (a drug that causes β-cell destruction resulting in an animal model of type 1 diabetes) (139). In this study, streptozotocin was given to two-month-old male rats, and 23 days later, they were challenged with amphetamine, an indirect dopamine agonist. This period of hyperglycemia significantly reduced basal levels and amphetamine-stimulated dopamine levels (**Figure 2**) in the nucleus accumbens, a region of the brain involved with reward, demonstrating that hyperglycemia significantly diminishes limbic system function and reward (37).

These results are particularly significant because the rats were comparatively young, with developing brains (based on level of myelination) comparable to 20-year-old humans, an age when the brain is fairly resistant to the pathologies of hyperglycemia and depression (140, 141). Over a third of patients with T2DM are 65 years or older, when the brain is far more susceptible to these pathologies. Hence, it is likely that humans with T2DM experience a more severe reduction in both basal and stimulated dopamine levels (142). Secondly, the period of hyperglycemia used in this study is less than needed to diagnose the binge eating disorder, which requires a 3-month duration (143). Considering that binge eating, i.e., consumption of a large amount of food in a short time, can reduce an individual’s insulin sensitivity and cause prolonged hyperglycemia, these data suggest that patients also experience hyperglycemia-induced limbic system inhibition. This inhibitory effect of hyperglycemia on the limbic system may initiate RDS and induce overeating, contributing to binge-eating behaviors (144).

In humans, T2DM has been shown to reduce the size of various limbic structures, which correlates with their reduced function (63, 64). Even though young age protects the brain from the atrophy commonly observed in depression, adolescent patients with T2DM have been shown to have significantly reduced hippocampus and prefrontal cortex volumes (145–149). This change occurred even though brain-derived neurotrophic factor (BDNF) levels in adolescents increase with BMI (150, 151), demonstrating that the impacts of T2DM on the brain are far worse than those typically observed in depression alone. In geriatric patients, T2DM has been shown to cause significant atrophy in the prefrontal cortex, amygdala, and hippocampus independent of vascular complications (152–154). In fact, declining cognitive function, including a higher rate of dementia, occurs in individuals with T2DM (155).

### Impact of T2DM on executive functions

In addition to altering the limbic system function, T2DM can also cause degeneration of the frontal lobe (which contains the prefrontal cortex) and the temporal lobes (which contains the amygdala and hippocampus), which has also been shown to impair executive functions and frequently results in disinhibition (156). This is characterized by overeating and an increased preference for sweet foods (156–159). Consistent with this, higher BMI correlated with reduced activation in the frontal cortex areas, implicated in inhibition (149). Another study found that frontal lobe volume predicted future weight gain in young women, where smaller volumes correlated with increased BMI one year later (160), demonstrating that T2DM-induced reductions in brain volume are associated with disinhibited eating and weight gain.

## Recommendations

Understanding the intimate connection between T2DM and depression, how they reinforce each other, and how they may induce food addiction and result in overeating is relevant to managing these conditions. A meta-analysis of comorbid depression and T2DM found that 31% of patients with T2DM suffer from depressive symptoms, with 11.4% having major depressive disorders, and the prevalence is significantly higher in females (161). Given that this comorbidity is associated with noncompliance to diabetes medications, increased diabetes complications, poor glycemic control, increased healthcare expenditures, and increased mortality (162–165), all patients with T2DM should be screened for depression (143). In an important study, Echeverry et al. (2009) examined the effects of administering the antidepressant sertraline (a selective serotonin reuptake inhibitor, SSRI) to low-income Hispanic and African Americans with poorly controlled T2DM and comorbid depression. They found that in addition to lowering their depressive symptoms, sertraline administration significantly reduced their HbA1c levels and systolic blood pressure (166). This could be explained by the increased adherence to diabetes recommendations in patients whose depression has improved by sertraline or by improvements in the HPA axis with the drug. Overall, patients with comorbid T2DM and depression benefit from interventions that target both conditions to see analogous improvements (167).

Our review has explained that hyperglycemia may play a role in the maintenance of binge-eating disorder, even in patients who are young and regardless of their diabetic status. Sodium-glucose cotransporter 2 (SGLT2) inhibitors, such as empagliflozin, inhibit glucose reuptake in the kidneys to lower blood sugar without causing hypoglycemia (168). For individuals with binge eating disorders that cannot be resolved by other therapies, SGLT2 inhibitors could allow them to maintain near normal blood glucose levels during binge eating episodes. Treatment with an SGLT2 inhibitor, although currently not approved by the FDA for binge eating disorders, could minimize many of this condition’s pathological impacts and prevent limbic system inhibition, which theoretically could reduce the frequency and duration of such episodes. However, more research is required to confirm this possibility. Interestingly, SGLT2 inhibitors are associated with a lower risk of dementia (169).

Nonpharmacological approaches commonly used for addiction should also be considered for patients with T2DM. Both epidemiological and mouse studies have consistently shown that the amount of aerobic exercise is inversely proportional to the use and abuse of addictive substances (170). Since eating itself is addictive, and T2DM further inhibits the limbic system amplifying this reward deficiency (described above), it can be inferred that increased activity will reduce overeating and benefit individuals with T2DM, regardless of caloric consumption. In fact, psychologists have appreciated for some time that any activity or hobby (variously known as “positive addictions” or “positive psychology”) would be beneficial in treating addiction (171, 172). This explains the widespread use of diverse activities, such as yoga and gardening, for mental health interventions and addiction treatments (173, 174).

Finally, individuals should avoid consuming foods containing high fructose corn syrup, which activates the limbic system, making it more likely to induce RDS and initiate or perpetuate food addiction (175).

## Conclusions

The ideal treatment of any disease is to target its underlying cause, not just its symptoms. In this article, we reviewed the evidence that eating, especially high-caloric foods, stimulates the limbic system, initiating RDS as the limbic system adjusts to and limits the stimulation in a process known as tolerance, thus linking the behavior of obesity to its psychiatric component (depression). Additionally, limbic inhibition (depression) and insulin resistance (which can lead to T2DM) stem from the same molecular mechanisms and directly stimulate one another while inducing weight gain. In these positive feedback loops, each continually stimulates the other and further inhibits the limbic and executive functions, exacerbating RDS. Treating the symptoms of T2DM, such as lowering HbA1c, without addressing the underlying pathways has little chance of eliminating the disease. Future research on the potential benefits of the six pillars of lifestyle medicine, which encompasses better diet, increased exercise, improved sleep, stress reduction, avoidance of risky behaviors, and fostering better relationships (176), could explore the impact of these lifestyle interventions on the shared pathways involved in conditions like T2DM, chronic inflammation, and depression.

## Author contributions

JW prepared the first draft of the manuscript, KL reviewed and revised the first draft, TF, AB, and ME reviewed the edited the revised manuscript. All authors contributed to the article and approved the submitted version.
